# Pan-immune-inflammation value is associated with poor prognosis in patients undergoing peritoneal dialysis

**DOI:** 10.1080/0886022X.2022.2158103

**Published:** 2023-01-12

**Authors:** Fengping Zhang, Luohua Li, Xianfeng Wu, Yueqiang Wen, Xiaojiang Zhan, Fenfen Peng, Xiaoyang Wang, Qian Zhou, Xiaoran Feng

**Affiliations:** a Department of Nephrology, Jiujiang No. 1 People’s Hospital, Jiujiang, China; b Department of Nephrology, Affiliated Sixth People’s Hospital, Shanghai, China; c Department of Nephrology, The Second Affiliated Hospital of Guangzhou Medical University, Guangzhou, China; d Department of Nephrology, The First Affiliated Hospital of Nanchang University, Nanchang, China; e Department of Nephrology, Zhujiang Hospital of Southern Medical University, Guangzhou, China; f Department of Nephrology, The First Affiliated Hospital of Zhengzhou University, Zhengzhou, China; g Department of Medical Statistics, Clinical Trials Unit, The First Affiliated Hospital of Sun Yat-sen University, Guangzhou, China

**Keywords:** Peritoneal dialysis, prognosis, pan-immune-inflammation value, biomarker

## Abstract

**Background:**

Immune-inflammatory biomarkers (IIBs) have been shown to be correlated with prognosis in patients undergoing peritoneal dialysis (PD). In this study, we aimed to evaluate the relationship between a novel comprehensive biomarker, the pan-immune-inflammation value (PIV), and the prognosis of patients undergoing PD.

**Methods:**

We retrospectively analyzed data from a multicenter, large-sample PD database. PIV was calculated as (neutrophil count × platelet count × monocyte count)/lymphocyte count. The prognostic endpoints in this study were all-cause death all-cause, cardiovascular disease (CVD) and infection-related death. The Kaplan–Meier method, a Cox proportional hazards regression, Fine–Gray competing risk model, smooth curve, and subgroup analysis were used to analyze the independent relationship between PIV and the prognosis of patients undergoing PD.

**Results:**

A total of 2796 cases of PD were included, and the study population was divided into Tertiles 1, 2, and 3, according to the tertiles of baseline PIVs. After adjusting for multiple model factors, patients in the Tertile 3 group had a significantly higher risk of all-cause death, CVD death and infection-related death compared with patients with PIV in the Tertile 1 group. Interaction tests showed no positive correlations for subgroup parameters. Regarding all-cause death, compared with the lowest tertile, the multivariable-adjusted hazard ratios (95% confidence intervals) of the highest and middle tertiles were 1.55 (1.25–1.94) and 1.77 (1.43–2.19), respectively; PIV (log2 processing) was associated with 17% excess of mortality in the continuous model.

**Conclusions:**

A high PIV at baseline was significantly associated with an increased risk of deaths due to all-causes, CVD and infection in patients undergoing PD.

## 1. Introduction

Peritoneal dialysis (PD) remains a common renal replacement therapy for patients with end-stage renal disease (ESRD) worldwide. The overall survival of these patients remains suboptimal, even with substantial technological innovations in PD [1]. There are many causes for the increased risk of death in patients undergoing PD, one of which is the increase in systemic inflammation and immune dysfunction. Inflammation accelerates the progression of atherosclerosis, promotes vascular and heart valve calcification, and aggravates protein energy consumption in patients undergoing dialysis. The immune dysfunction further increases the risk of patients with cardiovascular disease (CVD) and infection, resulting in poor prognosis of patients undergoing PD [2–3]. The readily available immune-inflammatory biomarkers (IIBs) based on peripheral blood counts, which typically divide counts of proinflammatory cells (e.g., neutrophils, platelets, and monocytes) by lymphocytes, have attracted increased attention in recent years. Several studies have reported that the neutrophil/lymphocyte ratio (NLR), platelet/lymphocyte ratio (PLR), monocyte/lymphocyte ratio (MLR), and systemic immune-inflammation index (SII) are associated with a poor survival rate in patients undergoing dialysis. [4–7]. Unfortunately, these metrics only assess partial cell counts and exhibit some deficiencies, with low predictive value. Hence, new biomarkers must be identified for better prognostic stratification of patients undergoing PD.

The pan-immune-inflammation value (PIV) is designed based on the abovementioned deficiencies, and the calculation of neutrophil count × platelet count × monocyte/lymphocyte count is a novel, easily measurable, and comprehensive indicator reflecting the immune response and systemic inflammation in the body [8]. Existing cumulative evidence suggests that the PIV is important in the prognosis or treatment response of patients with colorectal cancer, melanoma, breast cancer, non-small cell lung cancer, antineutrophil cytoplasmic antibody-associated vasculitis, and hidradenitis suppurativa [9–14]. However, reports on the relationship between PIV and prognosis in patients undergoing PD are not available. Hence, in this study, we aimed to explore the association between PIV and prognosis in patients undergoing PD using multicenter, large-sample data analysis.

## 2. Materials and methods

**Figure 1.**
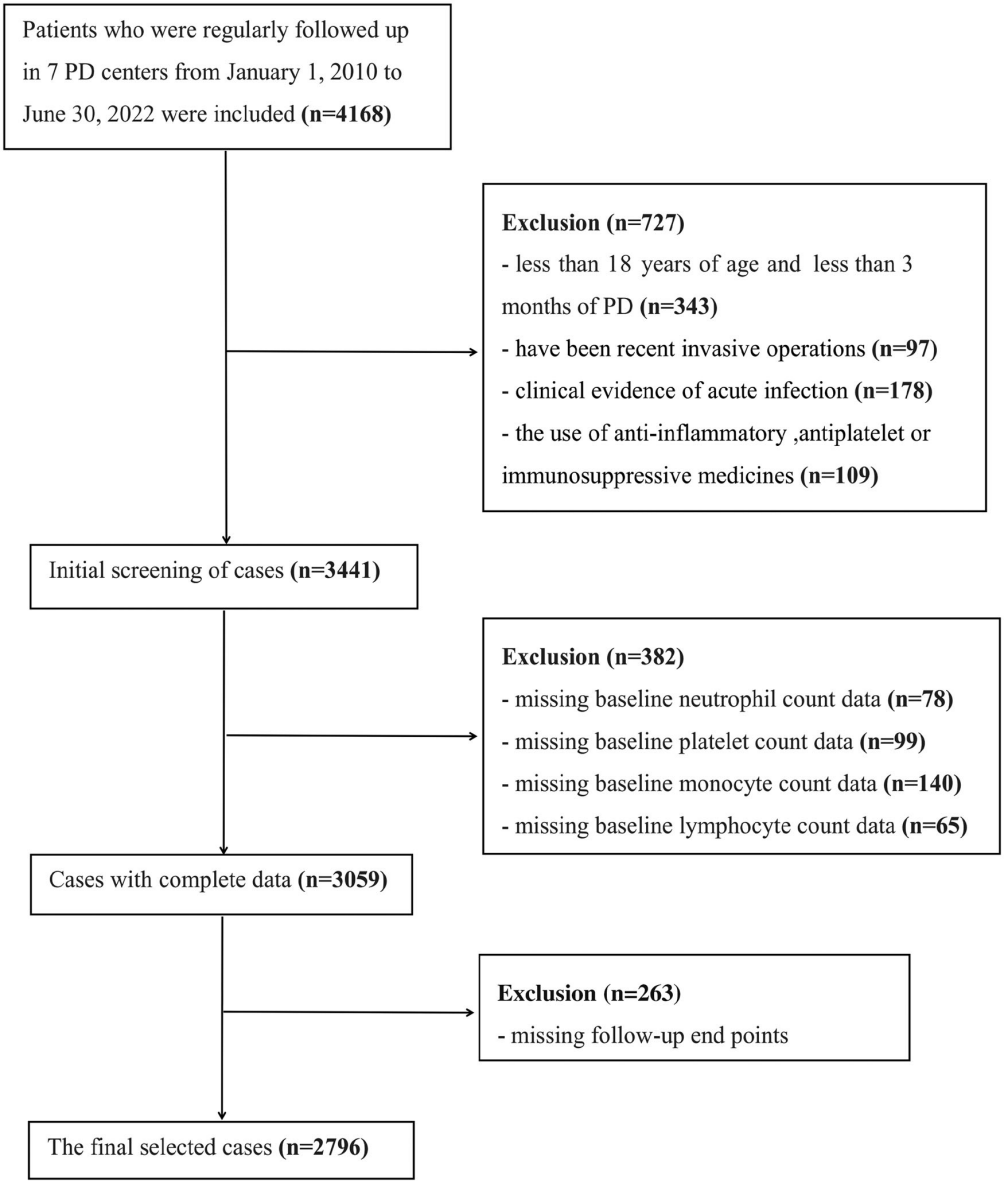
Flow chart of enrolled patients.

In this study, retrospective cohort data were collected from the Evergreen Tree Nephrology Association a large-scale PD database in China with complete and detailed records, designs, and dynamic updates. Patients with ESRD who started PD at PD centers in multiple regions of China were recruited. The original data registration and follow-up status classification of all cases was carried out according to unified standards, and the database was managed and maintained by specialized personnel. We examined the nutritional status, clinical adverse events, clinical outcomes, and prognosis of patients undergoing PD in southern China. This study initially included 4168 cases, however, 2796 cases were enrolled in the final analysis after the adoption of inclusion and exclusion criteria. The inclusion criteria for patient enrollment were as follows: (1) patients aged 18 years or older; (2) patients who had been on peritoneal dialysis for at least three months; and (3) ethnic Han Chinese. The exclusion criteria were as follows: (1) without baseline blood cell count data; (2) without outcome data; (3) patients who have been recent invasive operations; (4) clinical evidence of acute infection; and (5) the use of anti-inflammatory, antiplatelet drugs, or immunosuppressive drugs. **(**Figure 1**)**. It should be noted that the baseline data (within a week before PD) were collected before peritoneal dialysis catheterization. This study was approved by the local institutional ethics committee (jjsdyrmyy-yxyj-2021-107), which waived the need for written informed consent because this study was an observational study of case data, and no privacy issues were involved.

### 2.1. General data

We collected basic information about patients undergoing PD such as sex, age, body mass index, urine output, primary disease in ESRD, comorbidity history, and blood pressure; baseline laboratory data including complete blood count, aspartate transaminase, alanine aminotransferase, albumin, total cholesterol, triglyceride, high-density lipoprotein cholesterol, low-density lipoprotein cholesterol, fasting blood glucose, serum creatinine, uric acid, serum calcium, serum phosphorus, intact parathyroid hormone, C-reactive protein (CRP), and estimated glomerular filtration rate (eGFR).

#### 2.1.1. IIBs

Systemic inflammatory markers in this study were assessed using blood count data obtained at baseline. The formula for calculating PIV was a neutrophil count (10^9^/L) × platelet count (10^9^/L) × monocyte count (10^9^/L)/lymphocyte count (10^9^/L) [8]. The systemic inflammatory index (SII) refers to neutrophil count (10^9^/L) × platelet count (10^9^/L)/lymphocyte count (10^9^/L). The MLR was calculated as monocyte count (10^9^/L)/lymphocyte count (10^9^/L).

#### 2.1.2. Definition of study endpoints

The start time of data inclusion was January 2010, and the cutoff time of the study was June 2022. The endpoints of the study were CVD death and all-cause death. CVD death included heart failure, ischemic heart disease, pulmonary edema, arrhythmia, cerebrovascular disease, and other conditions. Other causes of death such as infections, tumor and malnutrition were defined as non-CVD death. The diagnosis of the cause of death was based on the medical records in the discussion of death cases.

### 2.2 Statistical analysis

In this study, measurement data are presented as mean ± standard deviation; the categorical parameters are presented as proportions, and continuous variables with a skewed distribution are presented as median (interquartile range [IQR]: P25, P75). The Kruskal–Wallis *H* rank-sum test was used to compare the differences in continuous variables between grouped data. The Mann–Whitney *U* test was used to compare the differences in non-normal continuous variables between grouped data. The chi-square (*χ*^2^) test was used to compare the differences in categorical variables between grouped data. The participants were divided into three groups according to the PIV tertile. Kaplan–Meier survival curves and log-rank tests were used to compare the differences in survival rates among the three groups. Multivariate fitting analysis and comparison of hazard ratios (HRs) were performed using a Cox proportional hazards model together with subgroup analysis. An interaction term was added to test for heterogeneity in the association between subgroups. The Fine–Gray competing risk model was used to analyze the risk factors of death owing to CVD in patients undergoing PD, with non-CVD as the competing risk. Smooth curve fitting was performed to investigate the overall association of the PIV at baseline with the risk of all-cause mortality. The net reclassification index (NRI) and integrated discrimination improvement (IDI) were used to assess the clinical utility of comparing the PIV with other IIBs. All analyses in this study were performed using R software (http://www.r-project.org) and Empower® (www.empowerstats.com; X&Y Solutions, Inc., Boston, MA, USA). *p* < 0.05 was considered statistically significant.

## 3. Results

### 3.1. Baseline characteristics of patients

Among the 2796 patients undergoing PD in this study, 1,274 were men and 1522 women, with mean age 52.6 ± 14.7 years. The study population included 2291 cases of hypertension, 598 cases of diabetes, and 795 cases of CVD history. The median (IQR) PIV of all patients at baseline (within a week before PD) was 272 (168, 424). According to baseline PIV tertiles, we divided patients undergoing PD into three groups: Tertile 1 (PIV <201, *n* = 932), Tertile 2 (PIV ≥201 and <362, *n* = 932), and Tertile 3 (PIV ≥362, *n* = 932). Among the three groups, there were statistically significant differences in age, sex, history of diabetes, history of CVD, and eGFR (*p* < 0.05), indicating that with increased PIV, the age, proportion of women, complications of diabetes mellitus, and CVD were also increased whereas the eGFR was decreased. However, PIV component elements and inflammatory-related indicators, such as neutrophils, platelet counts, monocytes, SII, MLR, and CRP levels were higher, and levels of lymphocytes and albumin were decreased with increased PIV. Table 1 shows the additional clinical, biochemical, and PD-related parameters of patients in this study.

**Table 1.** Baseline characteristics of peritoneal dialysis patients with different Tertiles of pan-immune-inflammation value (PIV).

	Total, *n* = 2796	Deficiency	Tertile 1, *n* = 932	Tertile 2, *n* = 932	Tertile 3, *n* = 932	*p*-Value
		*n* (%)	**(<201)**	**(≥201 and <362)**	**(≥362)**	
Age (years)	52.6 ± 14.7	–	49.7 ± 12.5	53.1 ± 13.9	55.0 ± 14.6	<0.001
Gender, n (%)		–				<0.001
Male	1,274 (45.6)		474 (50.8)	431 (46.2)	369 (39.6)	
Female	1,522 (54.4)		448 (49.2)	501 (53.8)	563 (60.4)	
Diabetes, *n* (%)	598 (21.4)	–	159 (17.1)	215 (23.1)	224 (24.0)	<0.001
Smoking, *n* (%)	485 (19.5)	308 (11.0)	169 (20.3)	163 (19.8)	153 (19.8)	0.534
Medications, *n* (%)						
Diuretic	522 (26.1)	274 (9.8)	182 (26.8)	162 (24.1)	178 (27.4)	0.201
β-blocker	246 (12.3)		82 (12.1)	79 (11.7)	85 (13.1)	0.612
Statins	412 (20.6)		127 (18.7)	136 (20.3)	149 (22.9)	0.064
CCB	1,417 (70.8)		487 (71.7)	471 (70.1)	459 (70.6)	0.723
Hypertension, *n* (%)	2,291(81.9)	–	754 (80.9)	761 (81.6)	776 (83.2)	0.400
Cardiovascular history, *n* (%)	795 (28.4)	–	238 (25.5)	254 (27.2)	303 (32.5)	<0.001
BMI (kg/m^2^)	22.0 ± 3.6	193 (6.9)	22.1 ± 3.4	22.0 ± 3.6	21.9 ± 3.5	0.467
Hemoglobin (g/L)	90.7 ± 14.3	–	91.3 ± 13.7	91.2 ± 14.2	89.9 ± 14.7	0.059
Neutrophil (10^9^/L)	4.4 ± 1.6	–	3.3 ± 1.2	4.3 ± 1.1	5.7 ± 1.7	<0.001
Lymphocyte (10^9^/L)	1.3 ± 0.7	–	1.4 ± 0.4	1.4 ± 0.6	1.2 ± 0.6	<0.001
Monocyte (10^9^/L)	0.5 ± 0.3	–	0.3 ± 0.1	0.5 ± 0.2	0.6 ± 0.3	<0.001
Platelet (10^9^/L)	196.5 ± 70.1	–	154.6 ± 54.5	209.1 ± 59.1	236.9 ± 73.4	<0.001
SII	592 (402, 889)	–	342 (258, 456)	594 (476, 771)	1002 (769, 1328)	<0.001
MLR	0.33 (0.24, 0.45)	–	0.24 (0.20, 0.31)	0.36 (0.27, 0.43)	0.48 (0.37, 0.61)	<0.001
ALT (U/L)	20.6 ± 7.1	112 (4.0)	20.6 ± 8.1	20.7 ± 7.0	20.4 ± 7.1	0.673
AST (U/L)	21.4 ± 6.3	112 (4.0)	22.1 ± 7.4	21.8 ± 6.1	21.9 ± 6.7	0.620
CRP (mg/L)	3.2 (1.1, 9.9)	289(10.3)	2.8 (1.1, 7.2)	3.0 (1.1, 9.6)	4.0 (1.3, 11.8)	0.008
Albumin (g/L)	35.9 ± 5.5	–	37.4 ± 5.2	36.0 ± 5.3	33.3 ± 5.4	<0.001
TC (mmol/L)	4.13 ± 1.52	262 (9.4)	4.01 ± 1.20	4.12 ± 1.63	4.16 ± 1.45	0.066
TG (mmol/L)	1.69 ± 0.49	262 (9.4)	1.67 ± 0.40	1.70 ± 0.44	1.69 ± 0.34	0.249
HDL-c (mmol/L)	1.28 ± 0.43	262 (9.4)	1.28 ± 0.31	1.30 ± 0.54	1.26 ± 0.45	0.152
LDL-c (mmol/L)	2.80 ± 0.56	262 (9.4)	2.80 ± 0.82	2.74 ± 0.84	2.83 ± 0.85	0.065
FBG (mmol/L)	5.43 ± 2.38	177 (6.4)	5.32 ± 2.75	5.34 ± 2.92	5.56 ± 3.31	0.161
Creatinine (umol/L)	762 (584, 971)	–	777 (584, 1002)	739 (582.9, 944.6)	746 (589, 975)	0.334
Uric acid (μmol/L)	408.9 ± 105.5	103 (3.6)	412.6 ± 116.4	405.8 ± 109.1	408.4 ± 104.9	0.405
Calcium (mmol/L)	1.94 ± 0.37	49 (1.7)	1.94 ± 0.42	1.95 ± 0.68	1.93 ± 0.25	0.671
Phosphate (mmol/L)	1.82 ± 0.44	52 (1.8)	1.79 ± 0.56	1.82 ± 0.32	1.83 ± 0.53	0.176
iPTH (pg/ml)	212 (115, 398)	167 (5.9)	238 (129.1, 419)	214 (114, 388)	195 (104, 376)	0.095
eGFR (ml/min/1.73m^2^)	6.2 (4.4, 8.2)	–	6.8 (4.4, 8.6)	6.4 (4.9, 8.0)	5.9 (4.1, 7.8)	<0.001

Abbreviations: CCB: calcium channel blocker; BMI, body mass index; SII: systemic inflammatory index; MLR: monocyte count/lymphocyte count; ALT: alanine aminotransferase; AST: aspartate aminotransferase; CRP: C-reactive protein; TC: total cholesterol; TG: triglyceride; HDL-c: high-density lipoprotein cholesterol; LDL-c: low-density lipoprotein cholesterol; FBG: fasting blood-glucose; iPTH: intact parathyroid hormone; eGFR: estimated glomerular filtration rate.

### 3.2. Relationship between PIV and risk of death owing to all causes and CVD mortality

**Figure 2.**
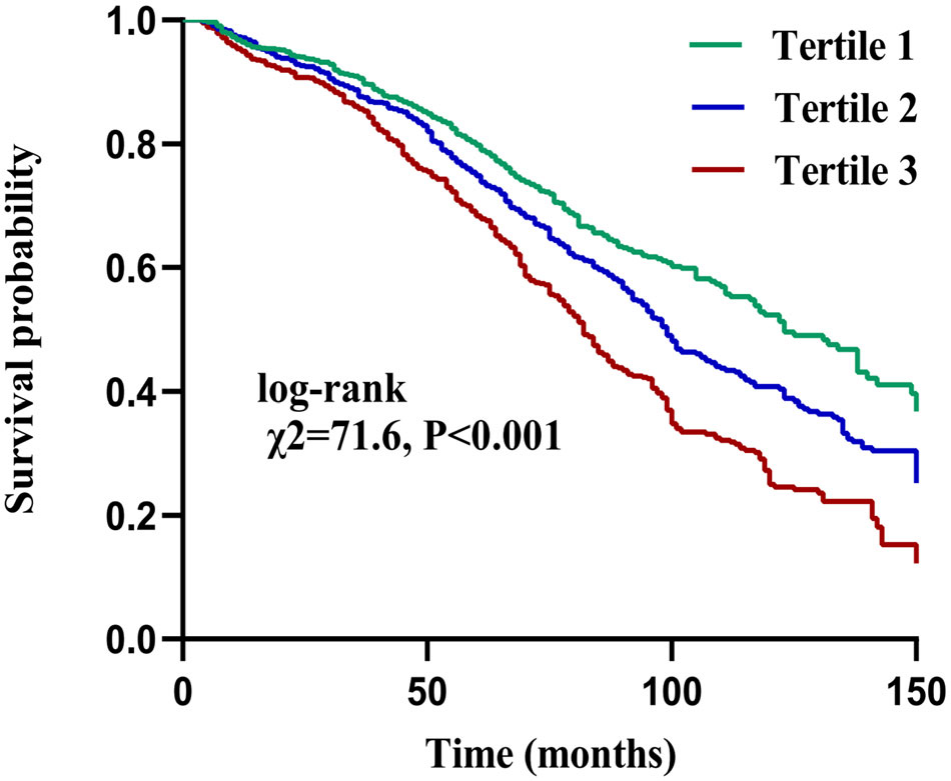
Kaplan–Meier curves of all-cause mortality in different pan-immune-inflammation value (PIV) tertiles.

The median (IQR) follow-up duration for all patients was 63 (34, 93) months, and 1158 patients had died by the end of the follow-up, including 599 (51.7%) deaths from CVD and 559 (48.3%) deaths from non-CVD. The results of the Kaplan–Meier survival curve showed that the overall survival rate of patients in Tertile 3 was significantly lower than that of patients in Tertiles 1 and 2 (log-rank test *χ*^2^=71.6, *p* < 0.001), as shown in Figure 2. We selected discrepant factors of univariate analysis, common demographic characteristics, and clinically controversial peritoneal dialysis deaths as covariates for Cox analysis. The results showed that, among continuous variables, increased PIV (after log2 processing) was associated with an increased risk of all-cause death [HR = 1.17, 95% confidence interval (CI): 1.04–1.31, *p* = 0.009]. Among categorical variables, Tertiles 2 and 3 had a significantly higher risk of all-cause mortality than Tertile 1 (HR = 1.55, 95% CI: 1.25–1.94, *p* = 0.017; HR = 1.77, 95% CI: 1.43–2.19, *p* < 0.001, respectively) **(**Table 2**)**.

**Table 2.** Cox regression analysis of pan-immune-inflammation value (PIV) and all-cause mortality.

	Model 1		Model 2		Model 3	
	HR (95% CI)	*p*-value	HR (95% CI)	*p*-value	HR (95% CI)	*p*-value
Continuous^a^	1.26 (1.14–1.39)	<0.001	1.28 (1.15–1.43)	<0.001	1.17 (1.04–1.31)	0.009
Categories						
Tertile 1	Reference		Reference		Reference	
Tertile 2	1.51 (1.24–1.80)	<0.001	1.54 (1.24–1.92)	0.005	1.55 (1.25–1.94)	0.017
Tertile 3	1.76 (1.44–2.05)	0.008	1.83 (1.48–2.26)	<0.001	1.77 (1.43–2.19)	<0.001
Trend. Test		<0.001		<0.001		0.004

^a^after log2 processing.

Model 1: no covariates were adjusted.

Model 2: was adjusted for age, gender, smoking, history of medication, history of cardiovascular disease, hypertension and diabetes.

Model 3: was adjusted for Model 2 + clinic factors (body mass index, hemoglobin, albumin, total cholesterol, low-density lipoprotein cholesterol, high-density lipoprotein cholesterol, C-reactive protein, calcium, phosphate, intact parathyroid hormone and estimated glomerular filtration rate).

**Figure 3.**
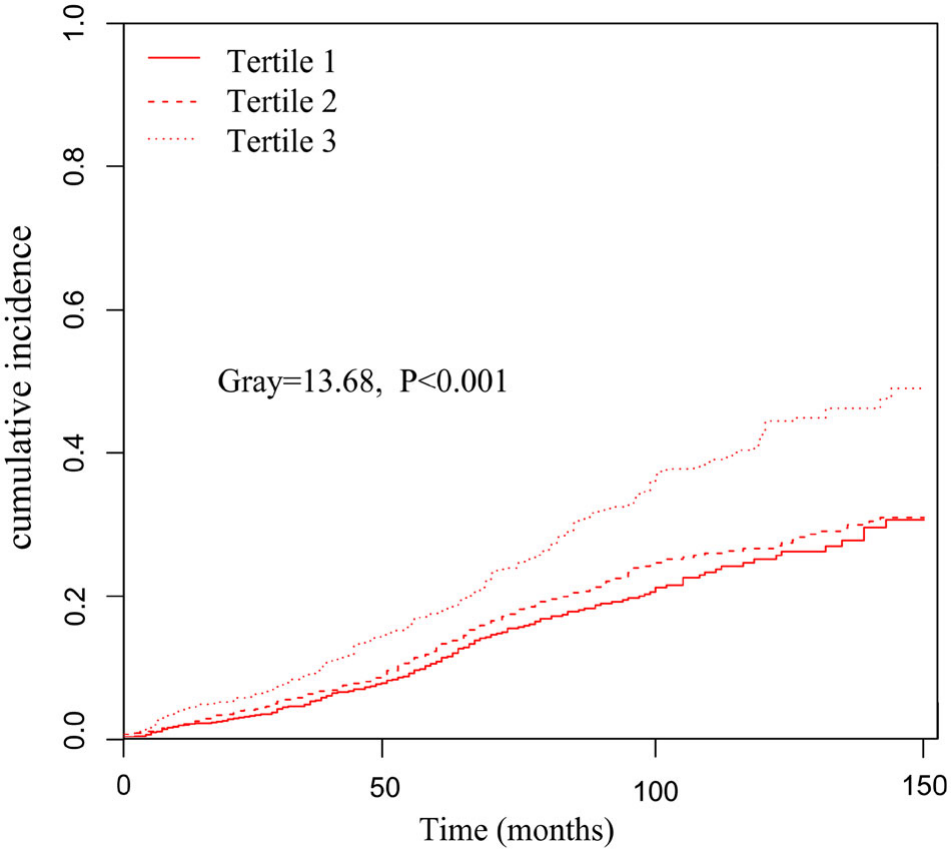
Cumulative event rates curves of cardiovascular disease mortality in different pan-immune-inflammation value (PIV) tertiles.

**Figure 4.**
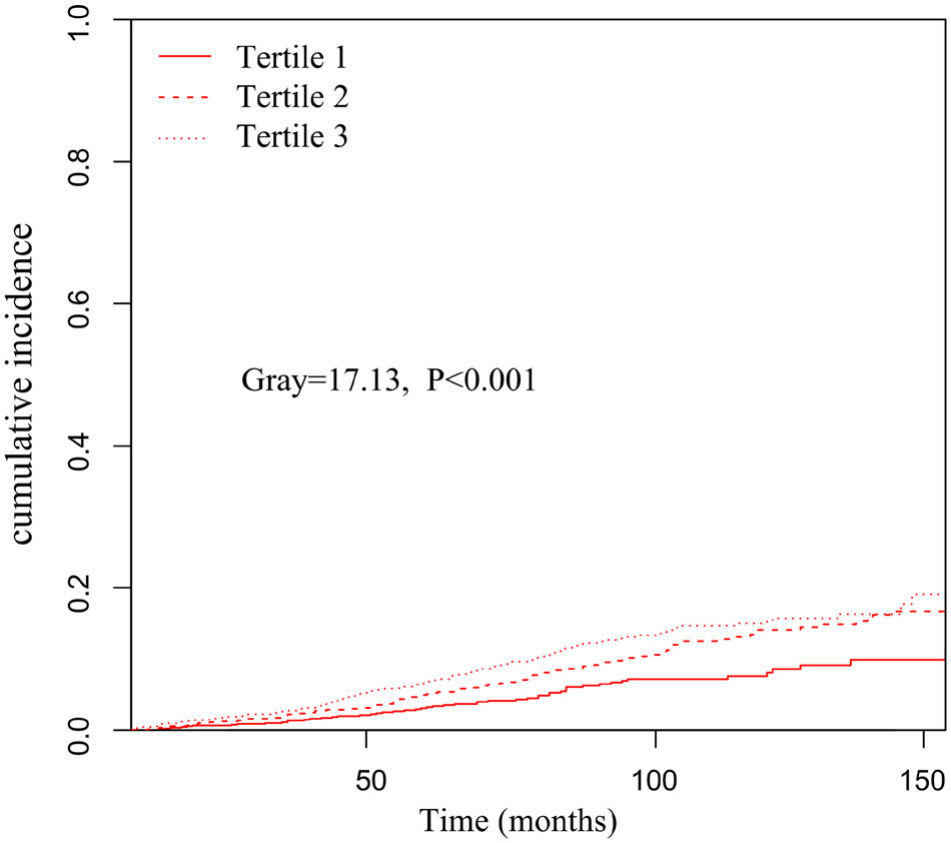
Cumulative event rates curves of infection-related mortality in different pan-immune-inflammation value (PIV) tertiles.

Further analysis using the Fine–Gray competing risk model showed that increased PIV was associated with increased death from CVD (Gray = 13.68, *p* < 0.001) **(**Figure 3). Simultaneously, a significant difference was also found after adjusting for multiple confounding factor models. Compared with the lowest PIV tertile, patients in the highest PIV tertile had a significantly 99% increased risk of CVD mortality [sub-distribution hazard ratio (SHR) =1.99, 95% CI: 1.40–2.83, *p* < 0.001] (Table 3). Meanwhile, we found the PD patients in Tertile 3 had a significantly higher incidence of infection-related mortality than Tertiles 1 and Tertiles 2 (Gray = 17.13, *p* < 0.001) (Figure 4).

**Table 3.** Association between pan-immune-inflammation value (PIV) and cardiovascular disease (CVD) mortality (Fine–Gray competing risk model).

	Model 1		Model 2		Model 3	
	SHR (95% CI)	*p*-value	SHR (95% CI)	*p*-value	SHR (95% CI)	*p*-value
Tertile 1	Reference		Reference		Reference	
Tertile 2	1.64 (1.22–2.17)	0.001	1.58 (1.17–2.23)	0.003	1.84 (1.30–2.59)	<0.001
Tertile 3	1.93 (1.44–2.59)	<0.001	1.73 (1.27–2.35)	<0.001	1.99 (1.40–2.83)	<0.001

Model 1: no covariates were adjusted.

Model 2: was adjusted for age, gender, smoking, history of medication, history of cardiovascular diseae, hypertension and diabetes.

Model 3: was adjusted for Model 2 + clinic factors (body mass index, hemoglobin, albumin, total cholesterol, low-density lipoprotein cholesterol, high-density lipoprotein cholesterol, C-reactive protein, calcium, phosphate, intact parathyroid hormone and estimated glomerular filtration rate).

**Figure 5.**
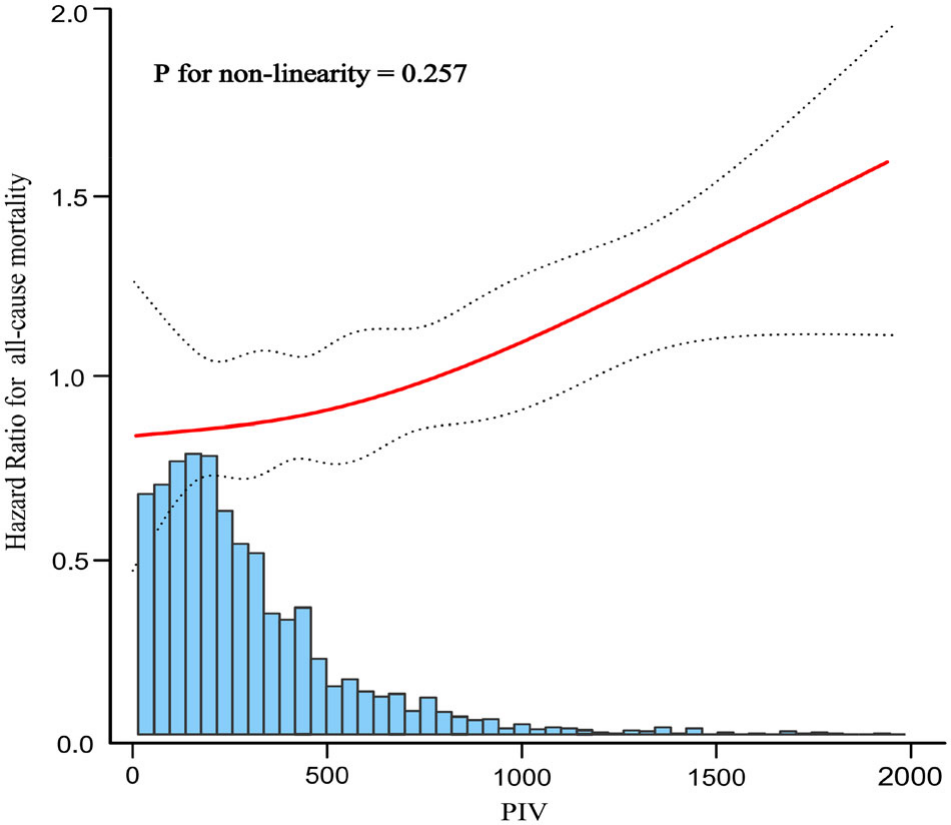
Curve fitting of pan-immune-inflammation value (PIV) and all-cause mortality after adjusted Cox analysis.

The smooth curve fitting graph was established and adjusted according to the covariates contained in Model 3. The linear relationship between PIV (raw values) and all-cause mortality was observed after adjusted Cox analysis (P for nonlinearity = 0.257) (Figure 5).

The solid red line represents the fitted curve and the dashed black line represents the 95% confidence interval; adjusted multivariable model was Model 3 in Table 2.

### 3.3. Subgroup analysis of PIV and risk of all-cause mortality

**Figure 6.**
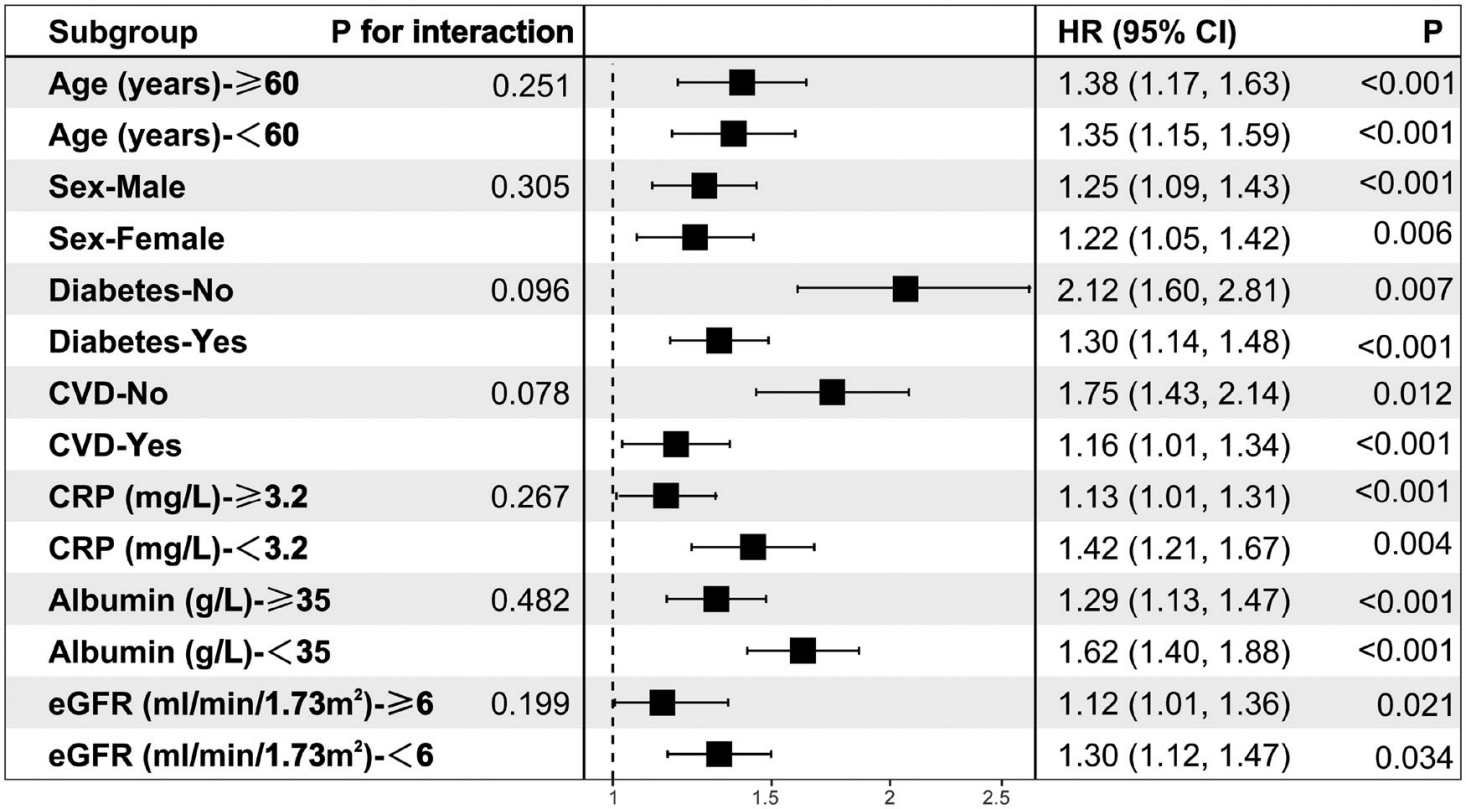
Forest plot for subgroup analysis of pan-immune-inflammation value (PIV) and all-cause mortality.

Additionally, we validated the results in age and sex subgroups and found that they were stable in all subgroups without interaction of specific subgroup analysis. The results showed that PIV was significantly associated with all-cause mortality in patients undergoing PD, after subgroups of patients based on their sex, age, diabetes history, CVD history, CRP level, albumin level, and eGFR (all *p* < 0.05). Although there was some inconsistency in specific HR values across subgroups, the correlation between PIV and all-cause mortality showed no significant difference between strata (*p* > 0.05 for all interactions), indicating that there was no significant dependence in age, sex, combined medical history, CRP level, albumin level, and eGFR among patients (Figure 6).

### 3.4. Comparison of PIV, SII, and MLR in predicting all-cause mortality

**Figure 7.**
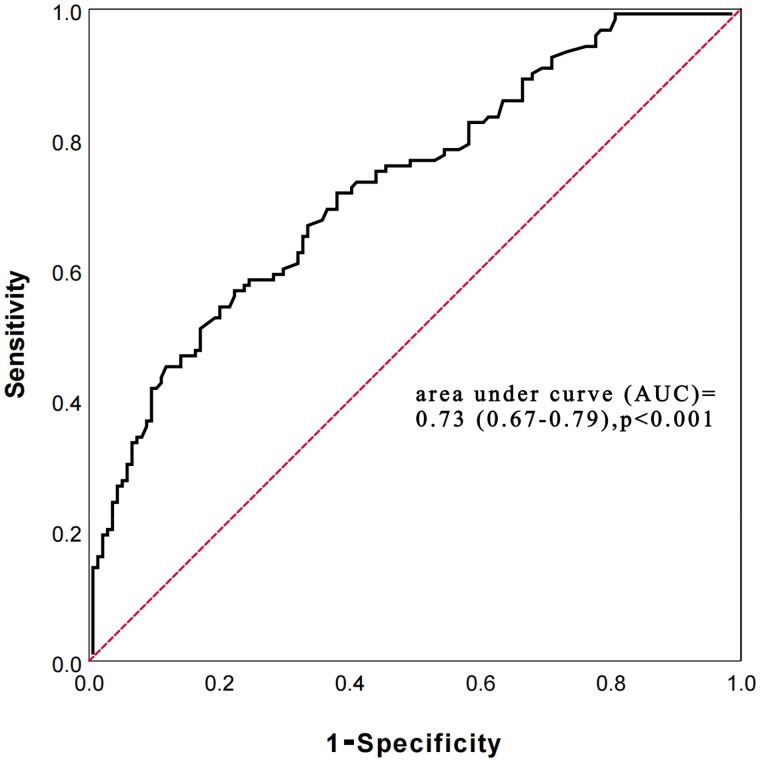
receiver operating characteristic (ROC) curve of pan-immune-inflammation value (PIV) in predicting all-cause mortality.

Receiver operating characteristic (ROC) curve analysis showed that the area under curve (AUC) of PIV for predicting all-cause mortality risk in PD patients was 0.73 (0.67–0.79), *p* < 0.001. The optimal cutoff value of PIV was 284, and the sensitivity and specificity were 72.7% and 64.6%, respectively (Figure 7).

The prediction accuracy of PIV with SII and MLR for all-cause death was compared using the difference in C-statistic, NRI, and IDI. The C-statistic of using PIV was 0.73 (0.69–0.77), *p* < 0.001, while the SII and MLR were 0.62 (0.54–0.70), *p* < 0.001 and 0.60 (0.52–0.68), *p* < 0.001, respectively. The delta C-statistics between PIV and SII was 0.11, *p* < 0.001, and between PIV and MLR was 0.13, *p* < 0.001. Compared with PIV, the NRI values of the SII and MLR were 0.38 (0.31–0.43), *p* = 0.019 and 0.49 (0.40–0.57), *p* < 0.001, respectively; the corresponding IDI values were 0.19 (0.15–0.24), *p* = 0.004 and 0.21 (0.16–0.26), *p* = 0.008 (Table 4). Positive NRI and IDI meant that the combined prediction value of PIV was higher than that of SII and MLR.

**Table 4.** C-statistic, NRI, and IDI of the PIV, SII and MLR in survival prediction.

Variable	C-statistic		delta C-statistics		NRI		IDI	
	Estimate (95%CI)	*p-*value	Estimate (95%CI)	*p-*value	Estimate (95%CI)	*p-*value	Estimate (95%CI)	*p*-value
SII	0.62 (0.54–0.70)	<0.001						
MLR	0.60 (0.54–0.67)	<0.001						
PIV	0.73 (0.69–0.77)	<0.001	^a^0.11 (0.04–0.17)	<0.001	^a^0.38 (0.31–0.43)	0.019	^a^0.19 (0.15–0.24)	0.004
			^b^0.13 (0.06–0.18)	<0.001	^b^0.49 (0.40–0.57)	<0.001	^b^0.21 (0.16–0.26)	0.008

Abbreviation: NRI: net reclassification index; IDI: integrated discrimination improvement; PIV: pan-immune-inflammation value; SII: systemic inflammatory index; MLR: monocyte count/lymphocyte count.

^a^ Compared with SII,

^b^ Compared with MLR.

## 4. Discussion

Using multi-center and long-term analysis of 2796 cases in this study, we showed for the first time that baseline PIV was linear related to the prognosis of patients undergoing PD. The Patients with high PIV had a poor prognosis. Multiple statistical analyses confirmed this high risk was not only for death due to CVD (Fine–Gray competing risk model, *p* < 0.001) but also for all-cause death (the previous statistics indicated that each unit of increased log2-PIV was associated with 17% increased risk of all-cause death). In particular, when we converted PIV to categorical variables, the SHR and HR of the highest PIV groups were significantly higher than the lowest. Subgroup analysis also confirmed that the association of PIV with prognosis was similar in different PD populations, suggesting that the correlation was independent of demographic characteristics, medical history, common clinical biochemical markers, and PD-related parameters. On the other side, we found that higher PIV was associated with infection-related mortality.

In recent years, PIV has come to be widely recognized as an inflammatory marker for various clinical diseases due to its comprehensive properties. A meta-analysis of 4942 patients with malignant tumors including a total of 15 studies showed that patients with higher PIV levels had a significantly higher risk of death than those with lower PIVs (HR = 2.00, 95% CI: 1.51–2.64, *p* < 0.001) [15]. Another study showed that the sensitivity and specificity of all-cause death in patients with antineutrophil cytoplasmic antibody-associated vasculitis were 52.0% and 71.2%, respectively (*p* = 0.041) when the cutoff value of PIV was set to 1011.3 [13]. Importantly, relative to the reported PIV values of healthy populations [206 (range, 52–1638)] [16]and patients with breast cancer [135.2 (IQR: 87.6–213.7)] [11], patients undergoing PD in this study seemed to have a higher baseline PIV, which may be related to the higher intensity of microinflammation in patients with ESRD.

The inflammatory stress burden is usually heavier in patients undergoing PD [17]. As important inflammatory cells, neutrophils have functions, such as phagocytosis, chemotaxis, and adhesion in patients with ESRD. Platelets are not only related to thrombosis, but also combine with endothelial cells to promote the migration and adhesion of leukocytes, triggering and aggravating inflammation in the body. In addition, monocytes reflect the function of macrophages, and the number of these pro-inflammatory cells usually reflects the inflammatory and immune status of these patients [18,19]. Azotemia and intestinal endotoxin permeability are increased in patients undergoing PD, resulting in disturbance of innate and adaptive immunity, inhibition of toll-like receptor 4 expression in monocytes, reduction of B-lymphocyte subsets, and decreased CD4+ T-lymphocyte count, leading to an increase in the infection rate [20]. Currently accepted evidence shows that systemic inflammation and immune dysfunction are directly or indirectly involved in the pathogenesis of atherosclerosis, CVD, and all-cause death in patients with uremia [21–22]. Thus, as an indicator that integrates the interaction between inflammation, platelet accumulation, and immunity, the PIV has a strong biological basis. It is not surprising that higher PIVs represent greater underlying inflammation and a poorer immune response capacity of the body, which is associated with all-cause death, CVD deaths and infection-related deaths in patients undergoing PD.

Previous studies have shown that demographic characteristics, such as age and sex, have a decisive influence on the reference range of data, such as IIBs [23–24]. We found similar characteristics in that patients with higher PIV were older and more likely to be female. To exclude the interference of these factors and ensure the integrity of our study, we adjusted for various covariates in Cox regression analysis. However, the HR value of the final adjusted model did not change significantly. The subgroup analysis and interaction test also showed that there was no significant interaction in the decisive effect of predicting all-cause death, regardless of the subgrouping of patient’s age, sex, combined medical history, CRP level, albumin level, or eGFR. No significant interaction dependence (*p* > 0.05 for all interaction) in the decisive effect of predicting all-cause death was found, suggesting that PIV has an independent and reliable value in the prognostic judgment of different populations of patients undergoing PD.

Although PIV is a new and available immune-inflammatory marker, there have been past analyses of IIBs, such as NLR, PLR, MLR, SII, systemic inflammation response index (SIRI) [25], and prognostic analysis of patients undergoing dialysis based on their improved blood counts. These indicators have not been extensively promoted due to the lack of parameters, limiting the prediction of body function and the general prediction ability, and resulting in inconsistent conclusions for subgroups. In this study, we compared the superiority of PIV with that of SII and MLR. Compared with PIV, SII, and MLR did not include the monocyte count and neutrophil count × platelet count, respectively. The C-statistic, NRI, and IDI results showed that PIV was better than SII and MLR in its comprehensive value regarding the prediction of death. It should be mentioned that the interplay between immunity, inflammation, and PD relies on a complex network. The PIV was created to incorporate multiple mediators in the immune-inflammatory system to more accurately model and reflect the inflammatory environment in patients undergoing PD and to prevent information about systemic inflammation from being fragmented. Because all pro-inflammatory cells in the blood count are included in the calculation, the PIV has better predictive power than incomplete IIBs. Similar to our viewpoint, the PIV has also been reported to have better predictive value than other IIBs in tumor-related research [8].

To our knowledge, this study was the first detailed analysis of the PIV and the prognosis of patients undergoing PD. We used a large sample size to conduct multi-aspect statistical analysis to verify the conclusions and subgroup analysis to assess the reliability of the data and correct for common confounding factors. We encourage the extended use of the PIV for predicting the prognosis of patients undergoing PD, especially given the availability of conditions for assessment. However, retrospective analysis along is insufficient. The medication history and laboratory data are not very complete. Selection bias is an inevitable disadvantage too. We could not find a causal relationship between PIV and death from PD. In addition, detailed data on PIV after PD initiation is not validated, so whether there will be significant fluctuations in PIV during PD and the impact of these fluctuations on poor prognosis is unknown. Multi-ethnic, prospective, complete and detailed follow-up PIV data, and controlled studies with more variables are still needed to verify the current findings.

## 5. Conclusions

PIV may better reflect the balance between immune and inflammatory pathways. A high PIV at baseline was associated with an increased risk of all-cause death, death owing to CVD and infection in patients undergoing PD, suggesting that the PIV is a novel, stable, and reliable biomarker for the prognosis of patients.

## Data Availability

The data presented in this study are available on request from the corresponding author (Xiaoran Feng). The data are not publicly available due to ethical and institutional reasons.

## References

[1] Li PK, Chow KM, Van de Luijtgaarden MW, et al. Changes in the worldwide epidemiology of peritoneal dialysis. Nat Rev Nephrol. 2017;13(2):90–103. 28029154 10.1038/nrneph.2016.181

[2] Li PK, Ng JK, Mcintyre CW. Inflammation and peritoneal dialysis. Semin Nephrol. 2017;37(1):54–65. 28153195 10.1016/j.semnephrol.2016.10.007

[3] Cohen G, Vanholder R. Special issue: immune dysfunction in uremia. Toxins (Basel). 2021;13(1):70. 33477769 10.3390/toxins13010070 PMC7832314

[4] Cai K, Luo Q, Zhu B, et al. Neutrophil-lymphocyte ratio is associated with arterial stiffness in patients with peritoneal dialysis. BMC Nephrol. 2016;17(1):191. 27881094 10.1186/s12882-016-0394-4 PMC5122148

[5] Liu S, Yang M, Zhao Q, et al. Platelet-to-Lymphocyte ratio is associated with the mortality in peritoneal dialysis patients. Iran J Kidney Dis. 2021;15(3):206–212. 33994380

[6] Wen Y, Zhan X, Wang N, et al. Monocyte/lymphocyte ratio and cardiovascular disease mortality in peritoneal dialysis patients. Mediators Inflamm. 2020;2020:9852507. 32214908 10.1155/2020/9852507 PMC7048939

[7] Ran Y, Wu QN, Long YJ, et al. Association of systemic immune-inflammation index with protein-energy wasting and prognosis in patients on maintenance hemodialysis. Zhonghua Yi Xue Za Zhi. 2021;101(28):2223–2227. Chinese. 34333935 10.3760/cma.j.cn112137-20210220-00445

[8] Fucà G, Guarini V, Antoniotti C, et al. The Pan-immune-inflammation value is a new prognostic biomarker in metastatic colorectal cancer: results from a pooled-analysis of the valentino and TRIBE first-line trials. Br J Cancer. 2020;123(3):403–409. 32424148 10.1038/s41416-020-0894-7 PMC7403416

[9] Pérez-Martelo M, González-García A, Vidal-Ínsua Y, et al. Clinical significance of baseline Pan-Immune-Inflammation value and its dynamics in metastatic colorectal cancer patients under first-line chemotherapy. Sci Rep. 2022;12(1):6893. 35477740 10.1038/s41598-022-10884-8 PMC9046216

[10] Fucà G, Beninato T, Bini M, et al. The Pan-immune-inflammation value in patients with metastatic melanoma receiving first-line therapy. Target Oncol. 2021;16(4):529–536. 34076798 10.1007/s11523-021-00819-0

[11] Lin F, Zhang LP, Xie SY, et al. Pan-immune-inflammation value: a new prognostic index in operative breast cancer. Front Oncol. 2022; 112:830138. 3 35494034 10.3389/fonc.2022.830138 PMC9043599

[12] Chen X, Hong X, Chen G, et al. The Pan-immune-inflammation value predicts the survival of patients with anaplastic lymphoma kinase-positive non-small cell lung cancer treated with first-line ALK inhibitor. Transl Oncol. 2022;17:101338. 34999541 10.1016/j.tranon.2021.101338 PMC8749135

[13] Lee LE, Ahn SS, Pyo JY, et al. Pan-immune-inflammation value at diagnosis independently predicts all-cause mortality in patients with antineutrophil cytoplasmic antibody-associated vasculitis. Clin Exp Rheumatol. 2021;129(Suppl):88–93. 10.55563/clinexprheumatol/m46d0v 33200738

[14] Gambichler T, Hessam S, Cramer P, et al. Complete blood collection-based systemic inflammation biomarkers for patients with hidradenitis suppurativa. J Eur Acad Dermatol Venereol. 2022;36(9):1593–1596. 35462426 10.1111/jdv.18175

[15] Guven DC, Sahin TK, Erul E, et al. The association between the Pan-Immune-Inflammation value and cancer prognosis: a systematic review and Meta-Analysis. Cancers (Basel). 2022;14(11):2675. 35681656 10.3390/cancers14112675 PMC9179577

[16] Gambichler T, Said S, Abu Rached N, et al. Pan-immune-inflammation value independently predicts disease recurrence in patients with merkel cell carcinoma. J Cancer Res Clin Oncol. 2022;148(11):3183–3189. 35098389 10.1007/s00432-022-03929-y PMC9508022

[17] Stenvinkel P. Inflammation in end-stage renal disease: the hidden enemy. Nephrology (Carlton). 2006;11(1):36–41. 16509930 10.1111/j.1440-1797.2006.00541.x

[18] Girndt M, Trojanowicz B, Ulrich C. Monocytes in uremia. Toxins (Basel). 2020;12(5):340. 32455723 10.3390/toxins12050340 PMC7290468

[19] Vaziri ND, Pahl MV, Crum A, et al. Effect of uremia on structure and function of immune system. J Ren Nutr. 2012;22(1):149–156. 22200433 10.1053/j.jrn.2011.10.020 PMC3246616

[20] Kelly CJ. T cell function in chronic renal failure and dialysis. Blood Purif. 1994;12(1):36–41. 7986474 10.1159/000170143

[21] Betjes MG. Immune cell dysfunction and inflammation in end-stage renal disease. Nat Rev Nephrol. 2013;9(5):255–265. 23507826 10.1038/nrneph.2013.44

[22] Dounousi E, Duni A, Naka KK, et al. The innate immune system and cardiovascular disease in ESKD: monocytes and natural killer cells. CVP. 2021;19(1):63–76. 10.2174/1570161118666200628024027 32600233

[23] Luo H, He L, Zhang G, et al. Normal reference intervals of neutrophil-to-lymphocyte ratio, platelet-to-lymphocyte ratio, lymphocyte-to-monocyte ratio, and systemic immune inflammation index in healthy adults: a large multi-center study from Western China. Clin Lab. 2019;65:3. 10.7754/Clin.Lab.2018.180715 30868857

[24] Meng X, Chang Q, Liu Y, et al. Determinant roles of gender and age on SII, PLR, NLR, LMR and MLR and their reference intervals defining in Henan, China: a posteriori and big-data-based. J Clin Lab Anal. 2018;32(2):e22228. 28378887 10.1002/jcla.22228 PMC6817185

[25] Li J, Li Y, Zou Y, et al. Use of the systemic inflammation response index (SIRI) as a novel prognostic marker for patients on peritoneal dialysis. Ren Fail. 2022;44(1):1227–1235. 35848372 10.1080/0886022X.2022.2100262 PMC9297720

